# Perspectives in Melanoma: meeting report from the Melanoma Bridge (December 2nd – 4th, 2021, Italy)

**DOI:** 10.1186/s12967-022-03592-4

**Published:** 2022-09-04

**Authors:** Paolo A. Ascierto, Sanjiv S. Agarwala, Christian Blank, Corrado Caracò, Richard D. Carvajal, Marc S. Ernstoff, Soldano Ferrone, Bernard A. Fox, Thomas F. Gajewski, Claus Garbe, Jean-Jacques Grob, Omid Hamid, Michelle Krogsgaard, Roger S. Lo, Amanda W. Lund, Gabriele Madonna, Olivier Michielin, Bart Neyns, Iman Osman, Solange Peters, Poulikos I. Poulikakos, Sergio A. Quezada, Bradley Reinfeld, Laurence Zitvogel, Igor Puzanov, Magdalena Thurin

**Affiliations:** 1 Department of Melanoma, Cancer Immunotherapy and Innovative Therapy, Istituto Nazionale Tumor IRCCS “Fondazione G. Pascale”, Naples, Italy; 2 Hematology & Oncology, Temple University and Cancer Expert Now, Bethlehem, PA USA; 3 Netherlands Cancer Institute, Amsterdam, Netherlands; 4 Division of Surgery of Melanoma and Skin Cancer, Istituto Nazionale Tumori “Fondazione Pascale” IRCCS, Naples, Italy; 5 Division of Hematology and Oncology, Department of Medicine, Columbia University Irving Medical Center, New York, NY USA; 6 Developmental Therapeutics Program, Division of Cancer Therapy & Diagnosis, NCI, Bethesda, NIHMD USA; 7 Department of Surgery, Massachusetts General Hospital, Harvard Medical School, Boston, MA USA; 8 Earle A. Chiles Research Institute, Robert W. Franz Cancer Research Center, Providence Cancer Institute, Portland, OR USA; 9 Department of Pathology and Department of Medicine (Section of Hematology/Oncology), University of Chicago, Chicago, IL USA; 10 Center for Dermato-Oncology, University-Department of Dermatology, Tuebingen, Germany; 11 Dermatology Department, Hopital de La Timone, Aix-Marseille, Marseille, France; 12 Medical Oncology, The Angeles Clinic and Research Institute, a Cedar-Sinai Affiliate, Los Angeles, CA USA; 13 New York Grossman School of Medicine, New York University Langone, New York, NY USA; 14 Jonsson Comprehensive Cancer Center David Geffen School of Medicine at UCLA, Los Angeles, CA USA; 15 Ronald O. Perelman Department of Dermatology, Department of Pathology, New York University Grossman School of Medicine, New York, NY USA; 16 Department of Melanoma, Cancer Immunotherapy and Innovative Therapy, Istituto Nazionale Tumori IRCCS “Fondazione G. Pascale”, Naples, Italy; 17 Precision Oncology Center and Melanoma Clinic, Oncology Department, Lausanne University Hospital (CHUV), Lausanne, Switzerland; 18 Medical Oncology, Universitair Ziekenhuis Brussel, Brussels, Belgium; 19 New York University Langone Medical Center, New York, NY USA; 20 UNIL, Medical Oncology Department European Thoracic Oncology Platform (ETOP), Specialized Thoracic Tumor Consultation, Oncology Department UNIL CHUV Thoracic Tumor Center, Lausanne University ESMO President, Scientific Coordinator, Lausanne, Switzerland; 21 Department of Oncological Sciences, Department of Dermatology Icahn School of Medicine at Mount Sinai, The Tisch Cancer Institute, New York, NY USA; 22 Cancer Immunology Unit, Research Department of Hematology, University College London Cancer Institute, London, UK; 23 Department of Medicine, Department of Medicine, Division of Hematology/Oncology Vanderbilt University Medical Center (VUMC), Graduate Program in Cancer Biology, Vanderbilt University, Nashville, TN USA; 24 Tumour Immunology and Immunotherapy of Cancer, European Academy of Tumor Immunology, Gustave Roussy, University Paris Saclay, INSERM, Villejuif Grand-Paris, France; 25 Department of Medicine, Roswell Park Comprehensive Cancer Center, Buffalo, NY USA; 26 Cancer Diagnosis Program, Division of Cancer Treatment and Diagnosis, NCI, Rockville, NIHMD USA

**Keywords:** Melanoma, Immunotherapy, Anti-PD-1, Anti-CTLA-4, Target therapy, Biomarkers, BRAF inhibitor, MEK inhibitor, Adjuvant, Neoadjuvant, Combination strategies

## Abstract

Advances in immune checkpoint and combination therapy have led to improvement in overall survival for patients with advanced melanoma. Improved understanding of the tumor, tumor microenvironment and tumor immune-evasion mechanisms has resulted in new approaches to targeting and harnessing the host immune response. Combination modalities with other immunotherapy agents, chemotherapy, radiotherapy, electrochemotherapy are also being explored to overcome resistance and to potentiate the immune response. In addition, novel approaches such as adoptive cell therapy, oncogenic viruses, vaccines and different strategies of drug administration including sequential, or combination treatment are being tested. Despite the progress in diagnosis of melanocytic lesions, correct classification of patients, selection of appropriate adjuvant and systemic theràapies, and prediction of response to therapy remain real challenges in melanoma. Improved understanding of the tumor microenvironment, tumor immunity and response to therapy has prompted extensive translational and clinical research in melanoma. There is a growing evidence that genomic and immune features of pre-treatment tumor biopsies may correlate with response in patients with melanoma and other cancers, but they have yet to be fully characterized and implemented clinically. Development of novel biomarker platforms may help to improve diagnostics and predictive accuracy for selection of patients for specific treatment. Overall, the future research efforts in melanoma therapeutics and translational research should focus on several aspects including: (a) developing robust biomarkers to predict efficacy of therapeutic modalities to guide clinical decision-making and optimize treatment regimens, (b) identifying mechanisms of therapeutic resistance to immune checkpoint inhibitors that are potentially actionable, (c) identifying biomarkers to predict therapy-induced adverse events, and (d) studying mechanism of actions of therapeutic agents and developing algorithms to optimize combination treatments. During the Melanoma Bridge meeting (December 2nd-4th, 2021, Naples, Italy) discussions focused on the currently approved systemic and local therapies for advanced melanoma and discussed novel biomarker strategies and advances in precision medicine as well as the impact of COVID-19 pandemic on management of melanoma patients.

## Introduction

Major advances have been made in the treatment of metastatic melanoma using immune checkpoint blockade, with the US Food and Drug Administration (FDA) approval of numerous therapeutic regimens within the past several years and many more being studied in clinical trials. Immunotherapies such as checkpoint inhibitors (cytotoxic T-lymphocyte-associated antigen [CTLA]-4 and programmed death [PD]-1/PD-ligand (L)-1 pathway) and targeted therapies have emerged as promising options. Furthermore, combination therapies including chemotherapy, immunotherapy (e.g., other immunotherapy agents targeting additional checkpoints such as lymphocyte-activation gene (LAG)-3, V-domain immunoglobulin suppressor of T cell activation (VISTA), T cell immunoglobulin and mucin-domain containing (TIM)-3, cytokines, vaccines, and oncogenic viruses), targeted therapy (e.g., BRAF, MEK) and radiotherapy have shown promising outcomes in efficacy and safety due to their multiple targets that may have synergistic effects on outcomes. Combinations of immunogenicity-inducing agents with immune checkpoint inhibitors are an especially promising modality of enhancing the endogenous antitumoral responses. These may help overcome primary resistance to anti-checkpoint monoclonal antibodies by recruiting T-cells to the tumor microenvironment (TME). Further advances in adoptive cell therapy such as chimeric antigen receptor (CAR)-T cells and combinations of anti-PD-1 therapies may also provide a successful treatment modality in patients with solid tumors.

Genomic and RNA-based studies exploring predictors of outcome to immune checkpoint blockade in melanoma suggest that tumor-specific mutational load and neoantigen signature as well as cytolytic activity are significantly associated with clinical benefit and increased overall survival (OS). For example, tumor mutational burden (TMB) demonstrates predictive value for response to immunotherapy that is based on enhanced neoantigen load and presentation to T cells. Although PD-L1 has been identified as a predictive marker in many tumors, its association with clinical efficacy in melanoma is yet to be proven. Other biomarkers based on transcriptomic profiles that reflect inflammation stratus, including tissue inflammation score, interferon (IFN)-α signature or CD8 T cell count, correlate with treatment response. Furthermore, biomarkers to predict immune-related adverse events (irAEs) are also of high interest, particularly those irAEs with life-threatening consequences. In addition to identifying predictors of response to immune checkpoint blockade, there is growing interest in understanding the mechanistic differences between different forms of immune checkpoint blockade. These advances should have important clinical implications and may help guide rational therapeutic combinations of distinct immune checkpoint inhibitors and immunomodulatory agents depending on the desired treatment effect.

## How COVID-19 impacted on our daily practice both clinical and research

### Clinical outcomes in cancer patients with COVID-19

Immunocompromised status secondary to malignancy and the use of immunosuppressive treatments may imply that patients with cancer are more vulnerable to infection with community-acquired respiratory viruses such as severe acute respiratory syndrome coronavirus 2 (SARS‐CoV‐2), and more prone to developing complications (e.g., lower respiratory tract illness and hypoxemic respiratory failure).

Several reports have suggested that patients with cancer are at increased risk of contracting SARS‐CoV‐2 and that they also experience worse outcomes. However, other data have indicated that patients hospitalized with coronavirus disease 2019 (COVID‐19) with or without cancer have similar outcomes when matched by age and number of comorbidities. The relationship between cancer status and survival outcome was assessed in hospitalized patients at NYU Langone Health who tested positive for COVID-19 during the height of the pandemic in New York [1]. A total of 6274 hospitalized patients were included, of whom 580 had either active cancer (n = 221; defined as treatment within 6 months of COVID-19 diagnosis or measurable disease at time of hospitalization) or a history of cancer (n = 359). Patients with history of cancer were significantly older than those with active malignancy and more likely to have comorbidities known to correspond with worse outcomes in COVID-19 (hypertension, atherosclerotic disease, and chronic kidney disease), as well as higher median body mass index.

However, COVID-19 associated morbidity was generally similar between the active cancer and history of cancer groups, with no significant differences in proportions with severe COVID‐19, admitted to intensive care, median length of hospital stay, or occurrence of thromboembolic events. There was a trend toward increased intubation in patients with a history of cancer, but this was not statistically significant. The two groups also received similar pharmacologic treatments and total number of medications for COVID‐19.

Despite similar morbidity, patients with active cancer had higher all‐cause mortality than those with a history of cancer (41% vs 32%; p = 0.035). This was even though patients with a history of cancer were significantly older and more often had a known risk factor for poor prognosis in COVID-19. Both active cancer patients and patients with a history of cancer had worse survival than the general population hospitalized with COVID-19 in New York City around the same time period.

These data highlight the importance of continuing cancer care with minimal interruptions during a pandemic to bring about response and remission as soon as possible. Routine management and monitoring should be complemented by encouraging the vaccination of active cancer patients and patients with a history of cancer.

### The experience at the melanoma unit of the istituto nazionale tumori—IRCCS “Fondazione G. Pascale” in Naples, Italy

The first rule during the COVID-19 pandemic is to protect patients and healthcare workers. Potential exposure to the virus must be minimized with the priority on the patient’s well-being. European Society for Medical Oncology (ESMO) guidance for the prioritization of cancer patients during the COVID-19 pandemic has been a tiered approach, with patients categorized as those requiring high, medium, or low priority intervention. One key recommendation has been that the treatment with targeted therapies or immunotherapies for patients with unresectable stage III or IV metastatic melanoma should be no stopped or delayed.

An ESMO multidisciplinary expert consensus on managing cancer patients during the COVID-19 pandemic concluded that treatment with immune checkpoint inhibitors should not be withheld or delayed in the absence of COVID-19 infection for the approved (neo)adjuvant treatment indications with demonstrated survival benefit [2]. However, in patients who have tested positive for COVID-19, immune checkpoint blockade should be postponed until recovery. One of the main concerns has been the possible increased risk of pneumonia in patients with cancer receiving immunotherapy. Interstitial pneumonia occurs in 2.5–5% of patients receiving immune checkpoint inhibitor monotherapy and 7–10% of patients receiving combination immune checkpoint inhibitor therapy and the risk of overlapping syndromes with similar pathogenesis in patients with COVID-19 is hypothetical but cannot be excluded.

In the study of 69 patients with lung cancer patients infected with COVID-19, PD-1 blockade was not associated with increased risk of severity of COVID-19 [3]. Similarly, an international, registry-based, cohort study of COVID-19 in 200 patients with thoracic malignancies reported no increased risk of hospitalization with immune checkpoint inhibitor therapy, either alone or combined with chemotherapy [4]. Moreover, preclinical data suggest that anti-PD-1 therapy can increase virus clearance. High pathological infection with influenza A is associated with increased PD-1 expression on influenza virus-specific CD8 + T cells in a mouse model, which is likely caused by the more inflamed airway microenvironment during the early days of infection [5]. PD-L1 inhibition in vivo led to reduced virus titers and increased CD8 + T cell numbers in high- but not low-pathological infection.

Few cases of infections secondary to the treatment of immune checkpoint inhibitor-related side effects have been reported and there are limited data describing viral infections or reactivations as a complication of immune checkpoint inhibitor use, even with the use of immunosuppressive treatment for irAEs [6]. Reports also show a decrease in virus replication in patients infected by hepatitis C virus, hepatitis B virus or human immunodeficiency virus who were on treatment with immune checkpoint inhibitors [7].

These data support the hypothesis that immune checkpoint blockade may be protective against SARS-CoV-2 infection. Data from the Istituto Nazionale Tumori—IRCCS "Fondazione G. Pascale" in Naples reported that immune checkpoint inhibitor treatment was a significant protective factor against the onset of COVID-19 infection as SARS-CoV-2 immunoglobulin (Ig)M and/or IgG titers were significantly lower among immune-checkpoint inhibitor treated patients than patients treated with chemotherapy [8]. However, ESMO guidance is that patients receiving immunotherapy showing signs of pneumonitis on computed tomography (CT) scan should be tested for COVID-19 before administrating steroids.

Treatment with high-dose steroids is the first-line treatment of irAEs with infliximab, mycophenolate mofetil, and tocilizumab options for steroid-refractory patients. Coronavirus infection activates pathways that result in increased systemic cytokine production, which contributes to the pathophysiology of severe COVID-19 infection. A potential therapeutic strategy is to target hyperinflammation, for example with interleukin (IL)-6 receptor antagonists such as tocilizumab, sarilumab or siltixumab. IL-6 is a cytokine with pleiotropic activity that plays an important role in acquired immune response by stimulation of antibody production and of effector T-cell development. Elevated IL-6 is a hallmark inflammatory signature seen in patients with severe COVID-19 respiratory distress. Early experience on the use of tocilizumab reported rapid improvement in both intubated and non-intubated COVID-19 patients. In the multicenter, single-arm TOCIVID-19 trial, tocilizumab reduced the lethality rate at 30 days without significant toxicity [9]. In a retrospective case series, clinical improvement after sarilumab treatment was associated with rapid decreases in C-reactive protein levels, as well as lower baseline IL-6 and neutrophil-to-lymphocyte ratio [10]. In addition to IL-6 inhibitors, several other therapies could potentially target hyperinflammation, including tumor necrosis factor (TNF) inhibitors, and inhibitors of IL-17, IL-23, IL-1, and Janus kinase (JAK). Further investigation of these as treatment options for COVID-19 is required.

### Impact of COVID-19 on oncology practice

The COVID-19 pandemic and non-communicable disease (NCD) epidemic, of which cancer is a significant part, have together brought about a deadly interplay. For example, 120 countries reported that NCD services were disrupted by COVID-19 and, in a more recent survey, several countries (US, Japan and Europe) all reported caseloads being reduced by between 25 and 51%. Fewer diagnosis, delays to surgery and chemotherapy, and switching treatment from intravenous to oral administration all occurred across the surveyed countries. Oncologists reported that patients were impacted on several levels, including financial hardship, logistical challenges due to new ways of working, appointment cancellations and delays, inadequate support and engagement, and reduced time with families.

COVID-19 has also had a major detrimental impact on cancer screening, with screening and accompanying diagnostic procedures significantly reduced. In the USA, an estimated 41,500 patients with breast cancer, 2000 patients with cervical cancer, and 24,000 patients with colorectal cancer had delayed diagnosis due to COVID-19. The impact of this is already being seen, with more patients having metastatic disease when first presenting to community oncologists in 2020 compared with pre-COVID. Decreases have also been seen in numbers of biopsies and surgeries, as well as in numbers of patients receiving systemic therapies.

Cancer is also a recognized risk factor for increased risk of severe illness from COVID-19. Across several surveys in Europe and North America, estimates of early mortality due to COVID-19 among cancer patients were 11–33%, with hematological and lung cancers associated with the highest risk. In a systematic review of 52 studies which included 18,650 patients with both COVID-19 and cancer, 4243 deaths were recorded giving a mortality probability of 25.6% [11]. COVID-19 fatality rate has also been shown to be higher in cancer patients than matched control groups of non-cancer patients.

COVID-19 has further highlighted major disparities in cancer care, with minority ethnic groups being more severely impacted in terms of access to diagnosis and treatments across countries with a disproportionate burden of cancer care disruptions.

Finally, COVID-19 had had a detrimental impact on oncologists, with high numbers reporting an adverse impact on their professional career and increased numbers considering leaving the profession. This impact has been experienced more by women than men, with a significantly higher proportion of women than men spending less time on science during lockdowns [12].

### VISTA as a target for immunotherapy

VISTA is an immune checkpoint regulatory molecule that is constitutively expressed on multiple immune cell types such as CD11b + myeloid cells, naïve CD4 + and CD8 + T cells, Foxp3 + CD4 + regulatory T cells (Tregs), and T cell receptor (TCR)γδ T cells. In murine models, treatment with VISTA antibodies increases the number of CD4 + and CD8 + tumor specific T cells in the TME and converts non-functional/exhausted CD8 + T cells to functional T cells [13, 14]. The CD8 + or CD4 + to Treg ratio was not increased in oral squamous cell carcinoma (SCC) but Tregs were decreased in melanoma. Myeloid-derived suppressor cells (MDSCs) were reduced in some but not all models. Heterozygous depletion of VISTA resulted in dermatitis, otitis, uveitis, and seizures.

In humans, high VISTA and low CD8 + expression predicted worse prognosis in primary oral SCC [15]. Increased CD4 + and CD8 + VISTA expression has been reported after ipilimumab therapy in patients with prostate cancer, with increased PD-L1 and VISTA inhibitory molecules on independent subsets of macrophages [16]. In breast cancer, CD4 + and CD8 + expressed VISTA [17] whereas in esophageal adenocarcinoma only CD4 + expressed VISTA; VISTA correlated with improved overall survival (OS) [18]. In non-small-cell lung cancer (NSCLC), VISTA was frequently expressed and was higher in T-lymphocytes than in macrophages. VISTA expression in the tumor predicted longer survival [19]. VISTA expression on MDSCs in patients with acute myeloid leukemia mediated the inhibition of T cell response [20].

In melanoma, increased expression of VISTA can indicate a poor prognosis. In an analysis of 85 primary melanoma specimens, VISTA was associated with myeloid infiltrate and the density of PD-1 + inflammatory cells [21]. The presence of VISTA was also associated with a significantly worse disease-specific survival in univariate and multivariate analysis.

Several clinical trials of anti-VISTA inhibitors have been conducted or are planned. The first trial of an anti-VISTA antibody, JNJ-61610588, in patients with advanced cancer was stopped early due to a dose limiting toxicity related to cytokine release syndrome. Studies of the small molecule VISTA/PD-L1 antagonist, CA-170, are ongoing, but single-agent therapy has shown limited efficacy. Phase I/II trials of anti-VISTA antibodies, including a fully human single-chain fragment variable (ScFv) anti-VISTA IgG1 monoclonal antibody designed to bind to VISTA through a unique epitope (KV12.1) and an IgG4 isotype anti-VISTA (HMBD-002), are planned. These trials will include anti-VISTA as monotherapy and in combination with anti-PD-1 therapy.

In conclusion, blocking VISTA antibody effects may depend on isotype and single-agent small molecule blockade has to date shown limited activity. The timing of anti-VISTA use is not yet established, and it is likely that combination immune checkpoint blockade will be needed.

## Melanoma is model for cancer research

### Microbiota-centered interventions for the next generation immuno-oncology

Primary resistance to immune checkpoint inhibition can be attributed to abnormal gut microbiome composition. Among patients with renal or lung cancer, metagenomics of fecal samples at diagnosis revealed enrichment of *Akkermansia muciniphila* taxa in patients responding to immune checkpoint inhibitor therapy [22]. In mice, oral supplementation with *A. muciniphila* after fecal microbial transplantation (FMT) from non-responders restored the efficacy of PD-1 blockade in an interleukin-12-dependent manner by increasing the recruitment of CCR9^+^CXCR3^+^CD4^+^ T lymphocytes into mouse tumor beds.

In the phase II randomized NEOSTAR trial of neoadjuvant nivolumab or nivolumab plus ipilimumab followed by surgery in 44 patients with operable stage III NSCLC, increased abundance of gut *Ruminococcus* and *Akkermansia* spp. that was associated with pathologic response to combination therapy [23].

### HLA class I defects in melanoma cells. molecular mechanisms and clinical relevance

Abnormalities in human leukocyte antigen (HLA) class I antigen-processing machinery (APM) have been identified in many types of cancer, with a frequency ranging between 36% in renal cancer and 88% in thyroid cancer for HLA class I heavy chains and between 17% in bone and soft tissue cancer and 73% in uveal melanoma for β-2 microglobulin [24] Multiple molecular mechanisms have been shown to result in defective HLA class I APM components expression and/or function in malignant cells (> 75%). Structural mutations are rarely the underlying mechanisms resulting in HLA class I APM defects which are frequently caused by epigenetic and dysregulatory mechanisms including dysregulated signaling, gene silencing by methylation, modification of chromatin structure by histone deacetylation, and inhibition by activated MAPK signaling pathway. Therefore, defects in HLA class I APM component expression can be corrected in most cases by strategies which counteract the underlying mechanisms.

### Evaluating the cancer surfaceome: a possible strategy to identify targets and monitor immunity

Both B cell and T cells are relevant in the immune response. B cell responses reveal antigens recognized by cytotoxic T lymphocytes and B cell immunity can have an anti-cancer effect. In a murine lung cancer model, neoantigen-driven B cell and CD4 T follicular helper cells promoted anti-tumor immunity by enhancing CD8 T cell effector functions [25]. Enrichment of T follicular helper cell and germinal center B cells correlated with favorable clinical outcomes. B cell-recognized antigens drive tumor-specific B cell and T follicular helper cell responses; tumor specific T follicular helper cells produce IL-21, which is critical for tumor control and tumor-infiltrating CD8 T cell effector function.

Mice vaccinated with a novel combination of an autophagosome-enriched vaccine derived from 4T1 mammary carcinoma and poly-I:C adjuvant demonstrate increases in antigen-specific CD8 + T cell recognition of 4T1 tumor cells and peptides. For proteins confirmed in 4T1 cells and vaccine by mass spectrometry, there is a correlation between increased CD8 + T cell IFN-γ release and vaccine-induced IgG, with both recognizing components of the same long peptide and recognition of both wild-type peptide and the neoantigen variant [26].

There is general agreement that cancer vaccines are ineffective as monotherapy. However, triple therapy in combination with a glucocorticoid-induced tumour necrosis factor receptor-related protein (GITR) agonist and PD-1 blockade can achieve sustained tumor effector T-cell responses in mice [27]. A clinical trial of triplet immunotherapy for head and neck squamous cell carcinoma (HNSCC) with vaccine (DPV-001) plus anti-PD-1 with or without anti-GITR has been initiated, with one of the primary aims being to assess T cell response to antigens relevant to HNSCC.

Proteogenomic analysis of major histocompatibility complex (MHC) class I-associated immunopeptidome of 19 primary acute myeloid leukemia samples identified 58 tumor-specific shared non-mutated non-canonical protein antigens [28]. These mainly resulted from epigenetic changes and intron retention and were coded by transcripts expressed in leukemic blasts and stem cells. As these genes were shown not to be expressed in the thymus, the host should lack central tolerance to these antigens, explaining the observation that these antigens elicited CD8 T cell responses in vitro and in vivo to these shared non-mutated cancer antigens. In another study, a proteogenomic method integrating ribosome profiling and mass spectrometry found that 2503 of 14,498 proteins identified in three human B cell lymphomas were non-canonical; 28% were new isoforms and 72% cryptic proteins [29]. Cryptic proteins are more disordered and unstable than classical proteins and are especially efficient at generating MHC-I peptides. It is possible that many of these proteins will not be available for cross-presentation when whole tumor cells are used as vaccines. Peptides originating from non-canonical proteins detected by ribosome profiling of malignant and healthy samples can be displayed on MHC-I of cancer cells, acting as additional sources of cancer antigens. Non-canonical proteins contributed 16% of all the detected proteins presented by the HLA complex in one study [30].

In summary, identification of novel non-canonical/unannotated proteins presented by HLA of human cancer cells generate many previously unrecognized cancer antigens. Many appear to be shared, lack central tolerance, and provide new opportunities for cancer vaccines and other immunotherapies. Better understanding of the function of these short-lived, rapidly degraded proteins provides new areas for research and potential novel therapeutic targets.

### Translational research (TR) for precision immuno-oncology in melanoma

Translational research can be used to guide precision immuno-oncology. Precision medicine involves integrating multiple data streams, including clinical data, genomics, and patient-reported outcomes, as well as newer techniques such as digital pathology and radiomics, into a clinical decision-making support tool. However, how to optimize output from this data collection remains a challenge.

Interrogating tissue at baseline may help allow identification of patients for the most appropriate treatment. Data can be derived from fresh tumor material and liquid biopsies. Fresh tumor processing techniques include single-cell RNA sequencing, fast analytical screening technique fine-needle aspiration (FAST-FNA), spatial transcriptomics, organoids, xenographs, and mechanistic studies. If there is no access to fresh source material, fixed tumor samples can be processed by immunohistochemistry, immunofluorescence, spatial transcriptomics, and digital pathology. Imaging techniques such as CT, magnetic resonance imaging (MRI), and positron emission tomography (PET) molecular imaging can also be employed by means of radiomics approaches. It is important that biomarkers can be identified using fixed tumor material because these can be more widely deployed in the clinic. These data can be used by a molecular tumor board to drive precision immuno-oncology in melanoma. Protocols aiming at maximizing the output from each translational sample are now available, with different strategies being adopted dependent on the volume of material available.

An example of translational research for precision immuno-oncology is provided by single-cell RNA sequencing. PEMSYS is an observational phase II trial designed to analyze modulation of the TME by pembrolizumab as first-line therapy in melanoma using a systems biology approach. Three single-cell tumor biopsies are taken per patient; at baseline, after 2 cycles of treatment, and at disease progression. Single-cell RNA sequencing is used to profile all tumor and microenvironment cells, with the current focus on cancer-associated fibroblasts (CAF). Clustering of CAFs reveals several subpopulations: myoCAFs and inflammatory/immune CAFs, which include immune-regulatory CAFs, antigen-presenting CAFs, and secretory (APOE +) CAFs. Plasticity has been observed between subpopulations. The Cancer Genome Atlas (TCGA) data deconvolution using CIBERSORTx, a machine learning method to infer cell-type-specific gene expression profile (GEP) without physical cell isolation, reveals an inverse correlation between immune-regulatory and antigen-presenting CAFs. Other CAF subtypes do not show a particular pattern. These data has been generated by Dr. Krisztian Homicsko from the Lausanne University Hospital.

On a larger scale, standardization of data is needed but sharing of -omics data has proven to be challenging. However, new opportunities are on the horizon. One example is ProjecTILs, an algorithm that aims to provide a common reference atlas for various single-cell RNA sequencing datasets [31]. ProjecTILs identifies gene programs that are altered in different conditions and tissues and the use of such strategies should help us share multi-omics data.

As fresh material management is quite complex, it is desirable to find correlated features in easily accessible data sources, i.e., fixed material and/or clinical images. Biomarkers based on digital pathology or radiomics would be highly desirable in the clinic. Interpretable machine learning algorithms for digital pathology show promise and could be used to select patient with increased benefit for a given immunotherapy.

**Fig. 1:**
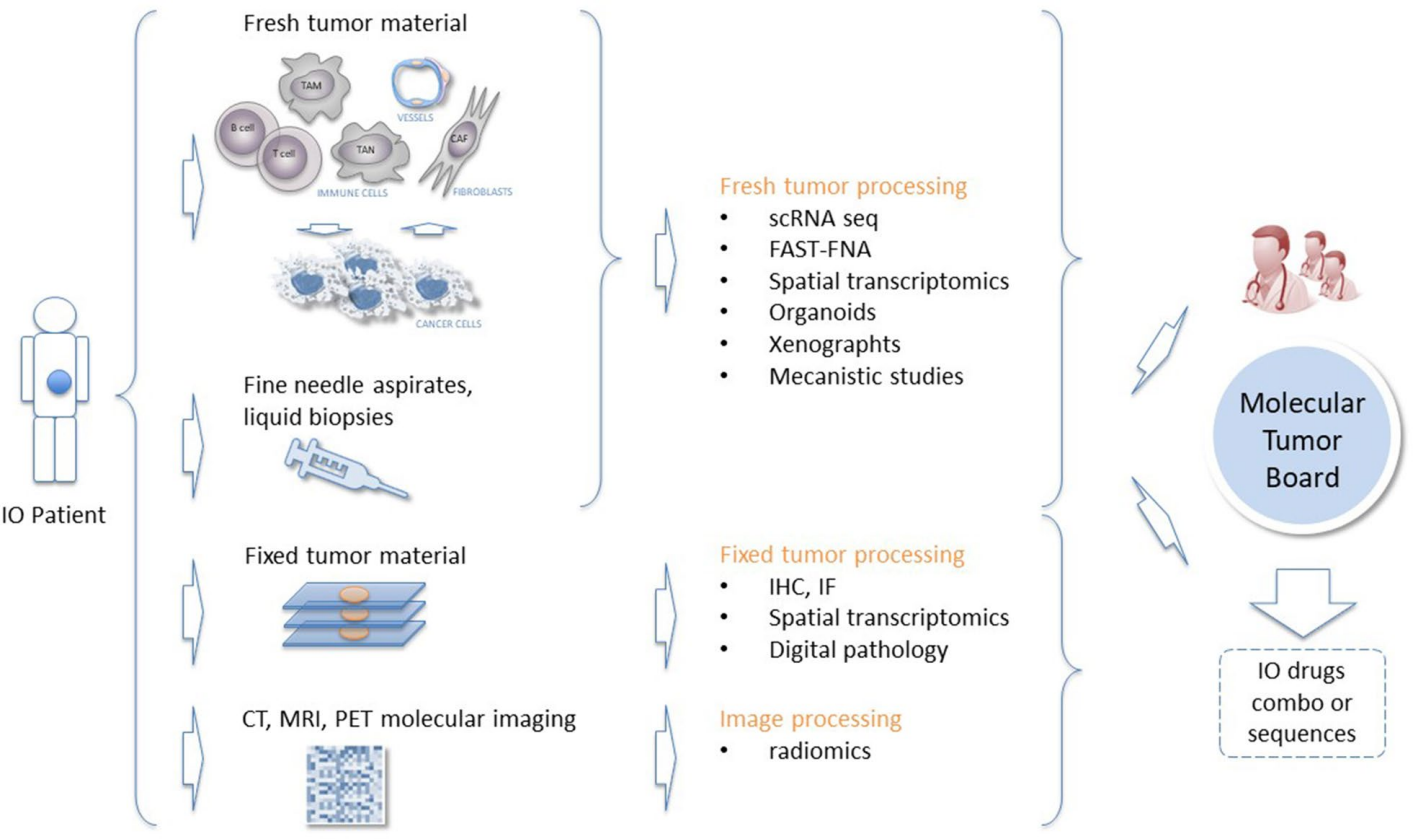
Possible melanoma TR strategy

In conclusion, translational research in cancer is fast evolving. New -omics strategies are providing unprecedented opportunities for biomarker development. Mechanistic studies remain a key to develop the causal relationships between tumor evolution, different cell subtypes, differentiation states of immune cells and response to treatment via multi-markers integration. Such studies represent powerful approaches to identify new targets and mechanisms of action of experimental immunotherapies to guide novel therapeutic interventions in cancer (Fig. 1).

### Gene expression profiling assays in the risk assessment of primary melanoma

GEP-based assays for melanoma are designed to predict recurrence or metastatic risk. There are currently three major GEP tests that have reported data in melanoma. The DecisionDX assay includes 31 genes with the endpoint of relapse-free survival (RFS) and prediction of sentinel lymph node biopsy (SLNB) metastases, the 8-GEP MelaGenix assay, which focused on melanoma-specific survival (MSS) in the adjuvant indication, and the 8-GEP CP-GEP test, which is prognostic for SLNB metastases combined with other clinic-pathological factors [32].

The three stages of GEP assay development are discovery, validation, and evaluation of clinical utility. The first step of discovery involves identifying genes, developing a training set, and establishing a formula with cut-off thresholds. Clinical validation involves evaluating performance for its intended use and its ability to discriminate between high and low-risk patients. Finally, the potential benefit for patient management needs to be evaluated, e.g., the selection of high-risk patients for adjuvant treatment.

In development of the MelaGenix assay, expression of 11 fresh frozen melanoma signature genes was quantified by reverse transcription polymerase chain reaction in a formalin-fixed paraffin-embedded (FFPE) melanoma training cohort [33]. A prognostic 8-gene signature in the tumor area (tumor and adjacent tissue) of stage I-III melanoma was identified. A signature based GEP score was calculated as the sum of the coded expression data of the genes and correlated with MSS. In a clinical validation cohort, the dichotomized GEP score discriminated significantly between short-term and long-term survivors and remained significant in a multivariate analysis that accounted for American Joint Committee on Cancer (AJCC) stage, age, and sex.

Utility of the 11-gene signature assay was tested in 245 patients with stage II melanoma using prospectively collected FFPE samples. Statistically significant survival differences were observed between patients with high and low GEP score for 10-year MSS, distant metastases-free survival (DMFS) and RFS [34]. Based on these data, the GEP assay is being used in an ongoing phase II adjuvant trial in patients with stage II melanoma who are SLN-negative within 12 weeks of SLNB. Patients with a high-risk GEP score are randomized to adjuvant nivolumab or observation, while low-risk GEP score patients undergo observation. The 11-gene GEP has also been assessed in 291 patients with stage I-III melanoma, with significant differences in 5-year disease-free survival (DFS) and MSS between patients with high and low-risk GEP scores [35].

In 2020, the US Melanoma Prevention Working Group concluded that, although every GEP test has prognostic power, their routine use is not supported at the current time. In the future, their use may help support identification of patients for SLNB and adjuvant therapy [32]. In particular, GEP may become important in identifying stage I-II patients suitable for adjuvant therapy and stage III patients in whom adjuvant therapy might be avoided.

### New insights into immunotherapy resistance revealed by Wilms tumor

The efficacy of anti-PD-1 therapy is greatest in T cell-inflamed tumors, characterized by a proinflammatory chemokine expression, infiltration of T cells, a type I IFN signature and immune escape via immune system-suppressive pathways. In comparison, immune resistance in non-T cell inflamed tumors is involves the exclusion of T cells, suppression of TME and disfunction of MHC among others [36]. Pediatric Wilms tumor displays the lowest tumor immune gene signature score of all human cancers. Investigation of Wilms tumor revealed an immune gene signature lower than matched normal kidney samples, other pediatric tumor samples, and adult kidney tumor samples, which reflects minimal CD8 + T cell presence [37]. Non-synonymous mutation frequency does not correlate with T cell gene signature in any cancers among TCGA, suggesting that mutation load and immune cell infiltration are independent events and that the absence of mutational neoepitopes is unlikely to explain the lack of a T-cell-inflamed TME in ‘cold tumor’ cancer types [38]. In Wilms tumors, a high DNA repair score inversely correlated with immune gene signature. A high DNA repair score also inversely correlated with the immune gene signature in most adult tumors in TCGA. In melanoma, DNA repair score inversely correlated with immune gene signature, and can be represented by DNA mismatch repair protein MSH2. Immunofluorescence staining for MSH2 inversely correlated with CD8 + T cells. High MSH2 expression was associated with poor CD8 + T cell infiltration and resistance to anti-PD-1 in metastatic melanoma.

Thus, investigating Wilms tumor revealed high expression of DNA repair enzyme genes linked to poor immune cell infiltration and activation. High MSH2 protein expression in melanoma is associated with anti-PD-1 resistance, which suggests the paradigm of inhibiting DNA repair in cold tumors to promote immunogenicity.

### Novel humanized mouse model to study mechanisms of immune-related toxicities

There is a causal relationship between pre-treatment auto-antibodies and irAEs. For example, baseline sera auto-antibodies from melanoma patients induced colitis in humanized Fcγ receptor (FcγR) mice treated with anti-PD-1. Melanoma patient IgG deposits were observed in colon and kidney tissues of humanized FcγR mice.

A novel humanized FcγR mouse model was developed for pre-clinical evaluation and prediction of irAEs to determine the effect of pre-existing autoantibodies. Purified IgG from melanoma patients with or without grade III-IV toxicity, as well as healthy subjects as control, were injected into humanized FcγR mice treated with anti-PD-1, anti-CTLA-4 or both anti-PD-1 and anti-CTLA-4. A colitis phenotype of humanized FcγR mice treated with immune checkpoint inhibitors was identified, which involved infiltration of the colonic tissue by CD8 + T cells, F4/80 + macrophage and Ly6 + neutrophils. GeoMx analysis confirmed the infiltration of colon in mice that developed colitis after transfer of toxic IgG and identified a colitis-specific gene signature with upregulation of immune cells expressing immune checkpoint inhibitors, such as LAG3, TIM-3, PD-1(PD-L1), and CTLA-4. Expression of VISTA and CD276 (B7-H3) as well as high levels of the T cell activation marker granzyme B were also observed. Distinct clusters of colitis-associated immune cells in mouse lamina propria were identified by single-cell RNA sequencing after anti-PD-1 treatment. Two distinct cell subsets were enriched in the colon of anti-PD-1-treated mice that developed a colitis phenotype: terminal effector CD8 cells (T-Ikzf2) and innate lymphoid cells group 3. T-Ikzf2 cells are a novel subpopulation not described previously in colitis. Some investigators have characterized cells with high levels of Ikzf2 and low expression of IL7 receptors as terminal effector CD8 + T cells, which fits a profile of the T-Ikzf2 identified in colons of mice developing colitis. Innate lymphoid cells have previously been linked to inflammation and are expected to be found in the inflamed colon. Microbial genomic profiling by 16sRNA sequencing showed that anti-PD-1 treatment causes a shift in the microbial composition, but this was not significantly different between experimental groups. Increased microbiome diversity with increased prevalence of *Verrucomicrobia* was observed in mice treated with anti-PD-1/anti-CTLA-4 combination; this creates disproportion in the microbiome and may be a prognostic factor for intestinal inflammation.

### Lymphatic transport and the intersection of melanoma immunity and metastasis

Melanoma tumor cells enter the lymphatic vessels and then the lymph nodes, which serve as a gateway in most early cancers. However, the lymphatic vasculature is not simply a conduit for metastases but is also the route for antigen presentation and dendritic cell (DC) transport and an active player in immune surveillance [39]. Lymphatic vessels regulate changes in intrinsic pumping and capillary remodeling and express a dynamic repertoire of inflammatory chemokines and adhesion molecules that facilitates leukocyte egress out of inflamed tissue. On arrival in lymph nodes, lymph orchestrates the rapid activation of adaptive immune responses.

The lymphatic system contributes to immune homeostasis and developing of melanoma through several overlapping and context-dependent mechanisms. Increased interstitial fluid load and inflammatory mediators influence intrinsic lymphatic pumping activity that contributes to local inflammation and anti-tumor responses.

Local inflammatory mediators act in coordination with lymphatic transport to affect tissue physiology and the kinetics of antigen presentation in lymph nodes. The lymphatic vasculature also regulates the migration of leukocytes, including DCs, to lymph nodes. DC migration is facilitated through chemokines and inflammatory cytokines, such as CCL21 and TNF-ɑ. The endothelial cells of the lymphatic vasculature also have their own intrinsic immunological properties, including the ability to cross-present antigen and the constitutive expression of checkpoint molecules, such as PD-L1 [40, 41]].

### A predictive model of analysis in real world data related to metastatic melanoma patients treated with immunotherapy

Outcomes with the real-world use of immune checkpoint inhibitors may differ from those observed in clinical trials. Because of this, there is a need to identify patients most likely to benefit from real-world treatment. The predictive and prognostic ability of several clinical variables were retrospectively investigated in 578 patients with metastatic melanoma treated with ipilimumab, nivolumab or pembrolizumab monotherapies at the Istituto Nazionale Tumori IRCCS Fondazione “G. Pascale”, Naples, Italy [42].

linical variables used to define patients’ risk of death or relapse and a weight given to each category was applied to create a e clinical categorization algorithm (CLICAL).. The weight of each variable and the number of the variables selected resulted in a predictive score which allows to determine the level of benefit to checkpoint therapy for melanoma patients.

The scores were grouped into predictive signatures from the worst benefit (signature I) to the best benefit (signature V). Included variables were age (younger vs. older), BRAF status (mutation vs. wild type), pretreatment with targeted therapy (yes vs. no), lactate dehydrogenase (LDH) (very high vs. high vs. normal), neutrophil-to-lymphocyte ratio (abnormal vs. normal), and eosinophil percentage (abnormal vs. normal). Signature V comprises a higher percentage of responder patients and a lower percentage of non-responder patients compared to signature I. Of interest, non-responders with higher signatures (IV-V) had better survival compared with non-responders with signatures I-III.

The algorithm was validated in an external cohort of 117 patients at Karolinska University Hospital, Stockholm, Sweden, The CLICAL algorithm identifies ied different groups of patients that differ in clinical outcome with the same benefit as in the l Italian cohort. The CLICAL was defined using a machine learning algorithm-survival random forest analysis topredict the response to checkpoint inhibitors therapy groups of melanoma patients..

**Fig. 2:**
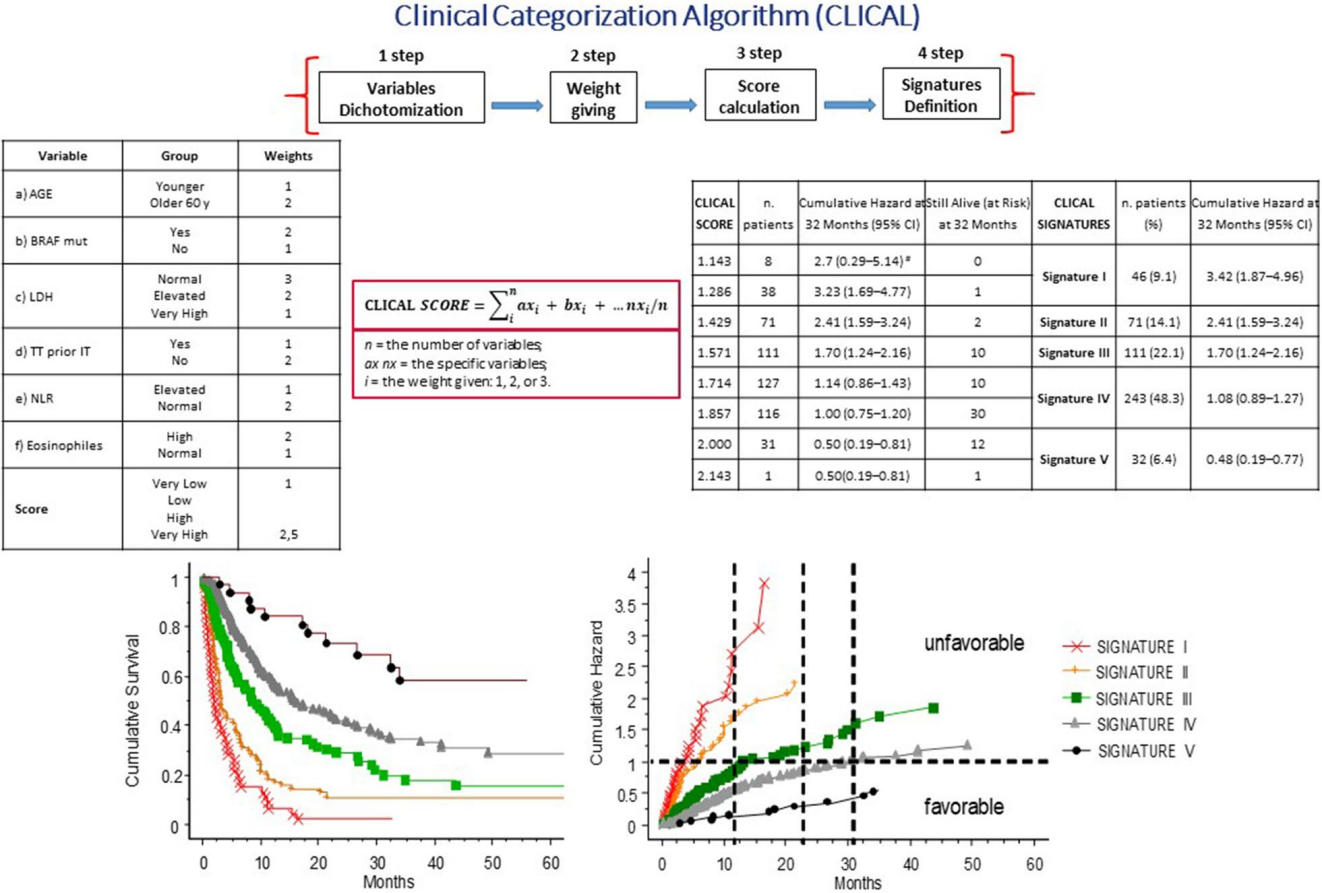
Clinical categorization algorithm

The use of a predictive algorithm has potential to identify patients likely to benefit from immunotherapy and so may support decision-making for patients referred for immune checkpoint inhibitor treatment. It is also being extended to cover more cancer types and datasets and has already been applied in colon cancer. A stand-alone web application is being developed (Fig. 2).

### The state of microbiome/fecal transplant

Numerous studies support a link between the gut microbiome and response to immunotherapy, so an important question is whether altering the microbiome in PD-1 refractory patients can result in antitumor responses.

Baseline gut microbiota affect the response to anti-PD-1 therapy; however, the species involved can differ. In a study in patents with melanoma treated with PD-1 checkpoint inhibitors, responders had increased *Firmicutes* phyla and increased bacterial diversity [43]. Other studies have reported increased *Bifidobacterium* spp, or *Akkermansia* spp. in responders [22, 44]. In patients with PD-1-refractory melanoma, Fecal Microbiota Transplant (FMT) from responders induced rapid and durable changes in the microbiota, with engraftment of bacteria belonging to *Firmicutes* and *Actinobacteria* phyla [45]. A single responder FMT resulted in engraftment in all responder recipient patients (n = 6/6) and some non-responders (n = 4/9). Recipient metagenomic changes were rapid, with the rate of compositional change slightly greater in responders. Profound induction of anti-commensal IgG was also seen in the serum of recipients, with the magnitude of change significantly greater in responders compared with non-responders.

Single responder FMT administration re-sensitized refractory melanoma patients to pembrolizumab. Responses included Response Evaluation Criteria in Solid Tumors (RECIST) responses and durable stable disease (> 12 months). Transplantation outcomes were similar whether donors were complete or partial responders; however, complete response donors had significantly greater bacterial diversity compared to partial response donors. In responders to FMT and pembrolizumab. adaptive T cell and memory immune responses were detectable peripherally; unbiased multiparameter flow cytometry from peripheral blood revealed that responders had greater CD8 + T cells which expressed high levels of activation markers and lower levels of CD27 CD56 + 8 + T cells which expressed high levels of activation markers, as well as increased T EMRA and mucosal-associated invariant T cells. FMT also reduced suppressive intratumorally IL-8-producing myeloid cells, with single cell RNA sequencing revealing that non-responders had higher frequency of myeloid cells which expressed CXCL8 (IL-8) following treatment. FMT produced dramatic shifts in the metabolome, with increased serum bile acids and more efficient transformation of primary to secondary bile acids in responders. Bacterial catabolism products of benzoate degradation pathways, associated with greater microbiome diversity, were higher in responders. In clinical trial in patients who relapsed following the checkpoint therapy FMT resulted in favorable changes in the TME and clinical responses.[46].

An oral rationally-defined bacterial consortium (VE800) of 11 clonal human commensal bacteria strains in combination with nivolumab is evaluated in a first-in-patient clinical trial in patients with selected types of metastatic cancer [47].

Composition of the gut microbiome arm may also influence the development of irAEs. In patients with metastatic melanoma treated with ipilimumab, increased *Bacteroidetes* phylum was correlated with less checkpoint-blockade-induced colitis, whereas *Ruminococcus* presence was associated with colitis [48]. A significantly higher abundance of *Bacteroides intestinalis* has also been observed in patients with a high rate of any grade ≥ 3 irAE, with upregulation of mucosal IL-1β in patient samples of colitis and in pre-clinical models [49].

### Perspectives in uveal melanoma

Systemic treatments commonly used to treat advanced cutaneous melanoma rarely achieve durable responses in patients with uveal melanoma, which is molecularly distinct from cutaneous and mucosal melanoma. Metastatic disease develops in approximately 50% of patients and is associated with poor survival due to the lack of effective treatment options. Thus, novel effective therapies are needed.

Uveal melanoma is characterized by mutations in GNAQ and GNA11, resulting in Ras/Raf/MEK/ERK pathway activation. Treatment with the MEK1/2 inhibitor, selumetinib, had limited efficacy either alone or with chemotherapy [50, 51]. However, combination approaches remain of interest, with another MEK1/2 inhibitor, binimetinib, demonstrating promising early activity in patients with metastatic uveal melanoma in combination with darovasertib, a protein kinase C inhibitor, according to preliminary data from an ongoing phase 1/2 trial [52]. Combined checkpoint blockade has also shown some degree of activity in uveal melanoma, with outcomes numerically superior to those seen with single agent therapy and should be prioritized outside of trial options for eligible patients [53].

Tebentafusp is a novel bispecific immune mobilizing T cell receptor-based agent with increased affinity for the glycoprotein (gp)100 peptide presented by HLA-A02 on tumor cells. In a phase III trial of 378 patients with previously untreated HLA-A*02:01-positive metastatic uveal melanoma, tebentafusp was well tolerated, with the most frequent adverse events consistent with its proposed mechanism of action [54]. The primary endpoint of OS was reached in the intention-to-treat population, with 1 year OS of 73% in the tebentafusp group compared to 59% in the control group (investigator's choice of therapy with single-agent pembrolizumab, ipilimumab, or dacarbazine) (HR for death, 0.51; p < 0.001).

The use of tebentafusp has now been approved by the FDA for the treatment of adults with HLA-A*02:01-positive metastatic uveal melanoma.

PFS was also significantly improved although not to the same extent as OS benefit. Tebentafusp-treated patients with any RECIST response, including best overall response of progressive disease, had survival curves trending above the control arm [55]. That an OS benefit with tebentafusp is observed even in patients with progressive disease suggests that RECIST-based radiographic assessments do not capture the complete benefit from tebentafusp.

Data from the phase II IMCgp100-102 trial in HLA-A0201-positive previously treated patients revealed 92% of patients had detectable ctDNA with almost all patients having mutations in known uveal melanoma oncogenes. Almost 70% of evaluable patients had a reduction in circulating tumor (ct) DNA on tebentafusp, and there was a linear relationship between magnitude of ctDNA reduction and improvement in OS. Reduction in ctDNA identified patients with OS benefit, regardless of best RECIST response, reinforcing the hypothesis that RECIST may under-estimate the OS benefit from tebentafusp.

HLA typing should be performed at time of metastasis for all patients and considered for patients with high-risk primary disease and tebentafusp should be offered to all eligible patients, whether previously treated or untreated. More research is needed to understand the disconnect between RECIST progressive disease and long OS with implied tumor burden decrease. Adjuvant and combinatorial strategies with tebentafusp are also areas that require urgent investigation. Unmet needs include HLA-A0201-negative patients and tebentafusp-resistant/refractory patients.

### Electrochemotherapy

Electrochemotherapy (ECT) involves the application of high intensity electric pulses to tissues to increase cell membrane permeability, allowing the direct diffusion of cytotoxic agents. In addition to its use in skin cancers, ECT can be employed for the treatment of deep-seated lesions, including liver, pancreatic, and prostate cancers, head and neck tumors, bone metastases, and gastrointestinal cancer. This has required new technological developments, such as deployable, expandable electrodes designed to access deep-seated target tissues. Targeting deep tumors also requires significant preoperative planning, with software being used to automatically generate the geometric configuration needed to guide electrode insertion and minimize the number of electrodes required while ensuring complete coverage of the target area.

Use of ECT should be integrated with other modalities, with indications including early cutaneous relapse after previous surgical treatment, complete or partial responses after previous ECT, palliation of hemostatic or painful lesions, and as neoadjuvant treatment of extensive lesions or to reduce surgical approach.

ECT can promote durable responses. In 60 patients with relapsed and refractory cutaneous melanoma metastases or in-transit disease, the objective response rate (ORR) of all treated lesions was 87% [56]. Thirteen patients (45% of complete responders) experienced a long-lasting response after one ECT session and were disease-free after a mean follow-up of 27.5 months. In analysis of prospective data from 28 centers across Europe collected over an 11-year period, including 987 patients with 2482 tumor lesions, the ORR was 85% [57]. ECT was effective across several cancer types, including malignant melanoma, basal cell carcinoma, breast cancer metastases, squamous cell carcinoma, and Kaposi's sarcoma. A higher complete response rate was observed for lesions < 3 cm in size, with linear array electrodes providing better tumor control than hexagonal electrodes in smaller lesions. In tumors > 2 cm, intravenous administration was superior to intratumoral administration.

ECT can produce an abscopal effect to enhance the benefit of combination with immunotherapy due to its immunogenic effect. In 127 patients treated with ipilimumab, the use of local irradiation, ECT, or selective internal radiotherapy of liver metastases prolonged OS [58]. Similarly, local ORR and PFS were higher with pembrolizumab plus ECT than with pembrolizumab alone (78% vs. 39%; p < 0.001, and 86% vs, 51%; p < 0.001, respectively) in a retrospective matched cohort analysis of patients with stage IIIC-IV melanoma [59]. Systemic disease control and survival were also increased with the addition of ECT.

## Emergent strategies

### Neoadjuvant in melanoma

Without systemic therapy, stage III melanoma patients have a poor prognosis, with a 5-year OS of only 30–60%. Even with adjuvant therapy, the RFS remains poor. However, neoadjuvant ipilimumab plus nivolumab was associated with better 4-year RFS and OS than an adjuvant ipilimumab plus nivolumab approach in the OpACIN study, with none of the patients with a pathologic response having relapsed [60]. In the OpACIN-neo trial which assessed dosing regimens of neoadjuvant ipilimumab plus nivolumab, the 2-year estimated RFS was 84% without adjuvant treatment. In an analysis of data pooled from six clinical trials of anti-PD-1-based immunotherapy or BRAF/MEK targeted therapy, pathologic response was shown to a better surrogate marker for immunotherapy than for targeted therapy [61].

In patients with clinical stage III or oligometastatic stage IV melanoma with RECIST v1.1 measurable, surgically resectable disease, neoadjuvant followed by adjuvant nivolumab plus the LAG-3 inhibitor relatlimab resulted in a pathological complete response (pCR) rate of 59% and near pCR rate (< 10% viable tumor) resulting in an overall major pathological response (MPR) rate of 66% [62]. All patients with a MPR were alive at one-year, versus 80% of patients without a MPR. The combination was well tolerated, with no treatment-related grade ≥ 3 in the neoadjuvant setting and no surgical delays due to treatment-related toxicity.

Previous studies have indicated that baseline IFN-γ signature high patients were more likely to respond to ipilimumab plus nivolumab. The DONIMI study will assess the combination of ipilimumab plus nivolumab combined with a class 1 histone deacetylase inhibitor, domatinostat, in 40 stage III melanoma patients with macroscopic de novo or recurrent disease according to IFN-γ signature [63]. Pathologic response rates were higher in in IFN-γ signature-high patients. At a median follow-up of 8.9 months, estimated 6-month RFS rate was 100% in IFN-γ signature-high patients and 79% in IFN-γ signature-low patients. Treatment was well tolerated. Several other neoadjuvant immunotherapy combinations in melanoma are being assessed in clinical trials. In conclusion, neoadjuvant therapy has the potential to reduce tumor burden and to provide prognostic information. Pathological response should be considered an early surrogate endpoint for clinical trials and a new benchmark for drug development and approval in melanoma.

### Exploring the activity of BRAF-/MEK-inhibition beyond currently approved indications

Plasma derived cell free (ctDNA) levels can be predictive of and improved clinical outcomes in patients with metastatic melanoma treated with PD-1 inhibitors. BRAF V600 mutant ctDNA could be useful as a biomarker and early predictor of acquired resistance in patients with BRAF V600 mutant melanoma.

In a phase II study a(cf)DNA test was used for the detection of BRAFV600 mutations. The of BRAF mutations in64.8%) of patients correlated with the results of the tissue test that had a positive BRAFV600 mutation with the cfDNA test [64]. In addition, 5.9% of patients with a wild-type BRAF status had a positive BRAF V600 mutant cfDNA test; retesting tissue confirmed BRAF V600 mutant status positivity in 5/7 patients.

. This indicates that cfDNA analysis may be used to confirm BRAF mutations status as an alternative to tumor biopsy. However, it should be noted that thesensitivity of cfDNA testing in this study was.

only 64.8%.

Resistance to BRAF-selective inhibitors can be reversible following treatment interruption, with responses shown in patients who progressed on BRAF V600 inhibitor therapy and who were rechallenged with dabrafenib and vemurafenib after treatment-free intervals of 4–8 months. [65]. In a phase II trial in patients with BRAF V600-mutant melanoma who had previously progressed on BRAF inhibitors (with or without MEK inhibitors) and were off-treatment for at least 12 weeks, dabrafenib plus trametinib rechallenge resulted in antitumor activity. with [66].

The mitogen-activated protein kinase pathway (MAPK-pathway) is frequently activated in melanoma by proto-oncogene driver mutations. MEK inhibitor monotherapy has activity in BRAF V600 wild-type, NRAS Q61R/K/L mutant melanoma and BRAF V600 wild-type/NRAS Q61R/K/L wild-type melanoma but is associated with considerable treatment-limiting skin toxicity. This can be mitigated by combining with BRAF inhibition. In a study of patients with advanced NRAS Q61R/K/L mutant melanoma pretreated with immune checkpoint inhibitors, trametinib plus low-dose dabrafenib mitigated MEK-inhibitor-related skin toxicity but clinical activity was insufficient [67]. However, promising antitumor activity, with an ORR of 43% in 14 evaluable patients and acceptable toxicity were observed in an interim analysis of NRAS Q61R/K/L wild-type patients [68].

**Fig. 3:**
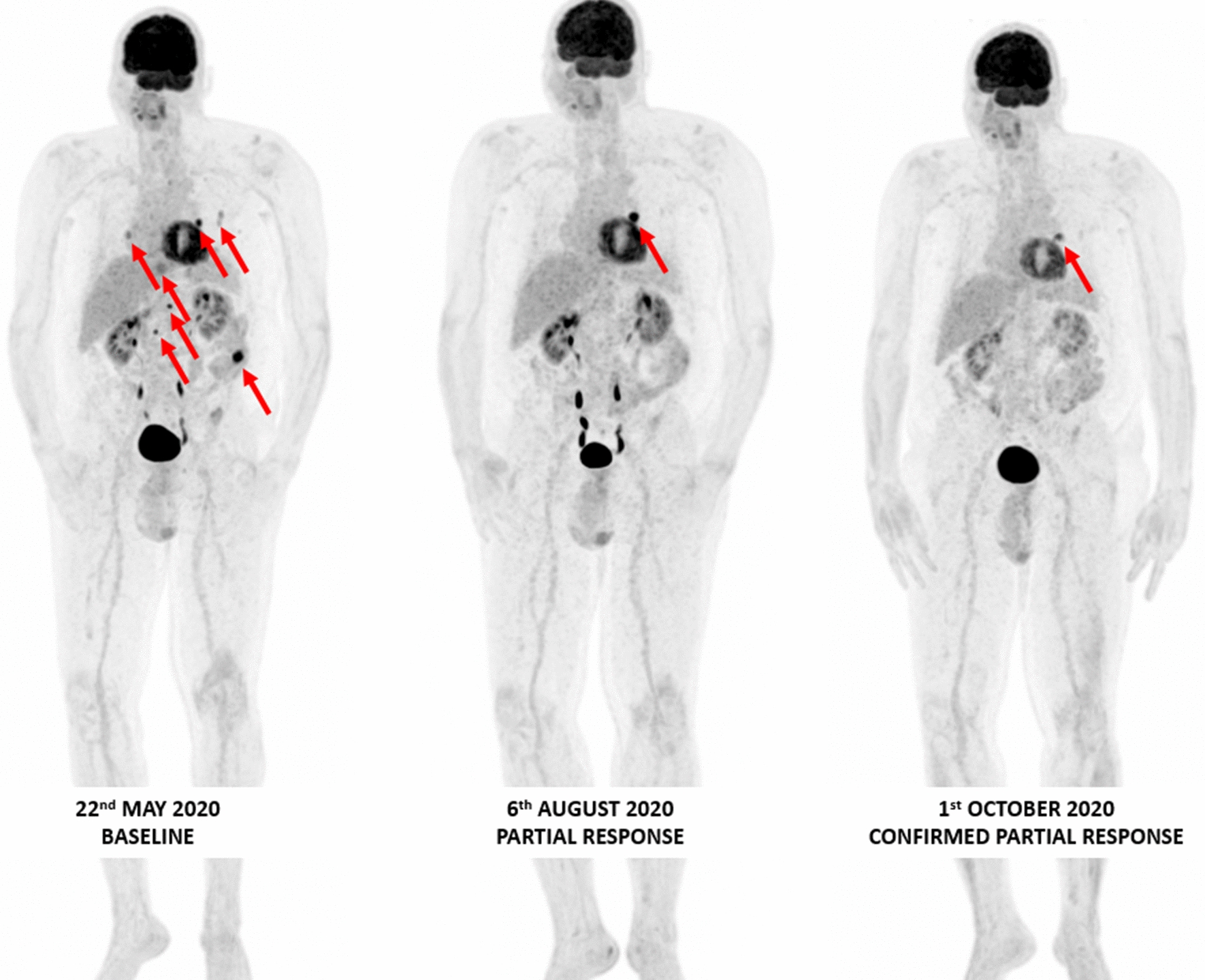
Confirmed tumor response on whole body 18F-FDG PET/CT MIP images obtained in a66 year old male stage IV-M1c melanoma patient with a BRAF N486_P590del (in-frame deletion, class II BRAF mutation) treated with trametinib 2 mg QD plus dabrafenib 50 mg BID

Future possibilities include the use of pan-RAF inhibitors and BRAF paradox breakers. Also, a class II BRAF inhibitor regorafenib, can seemingly reverse resistance to immunotherapy in colorectal cancer. A rapid clinical response was observed with off-label treatment with regorafenib plus low-dose trametinib in a heavily pre-treated stage IV NRAS Q61R-mutant melanoma patient. This approach is being investigated in a phase II 2-stage design trial investigating regorafenib in pretreated melanoma (RegoMel) (Fig. 3).

### Developing a new antibody targeting regulatory T cells in cancer

CD25 is the high affinity subunit of the IL-2 receptor-α that is highly expressed on Tregs and upregulated on activated T effector (Teff) cells in vitro. Scientific and pre-clinical study of CD25 have mostly focused on two main aspects of the receptor blocking that might be useful in treating autoimmunity and regulatory T cell depletion. In murine tumors, CD25 is primarily restricted to Tregs and not Teff cells while in humans it is preferentially expressed on tumor infiltrating Foxp3 + Tregs. Based on these data, CD25 was considered as a potential target for immunotherapy; however, lack of therapeutic activity of the anti-CD25 antibody PC61 in mice and humans limited further development. Lack of Treg depletion at the tumor site correlates with lack of protection and upregulation of the inhibitory FcγR IIb at the tumor site prevented intratumoral Treg cell depletion. Use of an Fc engineered anti-CD25 antibody with enhanced binding to activating FcγRs led to effective depletion of tumor-infiltrating Treg cells, increased Teff-to-Treg cell ratios, and improved control of established tumors [69]. This Fc optimized anti-CD25 antibody synergized with anti PD-1 to reject established tumors in mice.

Anti-CD25 antibodies (daclizumab, basiliximab) developed to prevent acute organ rejection and/or to treat multiple sclerosis block the IL-2/IL-2 receptor interaction, as does the anti-CD25 PC61 clone that is widely used for depletion of Treg cells in vivo.. However, In vivo activity of these Treg-depleting anti-CD25 antibodies is likely limited by their IL-2 blocking activity on the effector compartment. Increased effector response and antitumor activity may be induced by an anti-CD25 depleting antibody that does not affect IL-2 signaling.

An anti-CD25 antibody optimized to deplete Tregs whilst preserving IL-2-STAT5 signaling on effector T cells showed potent single dose, single agent antitumor activity across various mouse models [70]. This potent activity of non-IL-2 blocking anti-CD25 is reliant on the ability of effector cells to sense endogenous IL-2. In a poorly immunogenic melanoma mouse model, combination of non-IL-2 blocking anti-CD25 with Gvax vaccine induced tumor regression, delayed tumor progression and improved OS. The vaccine plus non-IL-2 blocking anti-CD25 drives Treg depletion/CD4^PD1^ high depletion, effector infiltration, and innate cell activation.

These data led to the development of the first human non-IL-2 blocking anti-CD25 (RG6292), which has been shown to preferentially deplete Tregs in PBMCs and human tumor samples. In a phase I dose escalation study, RG6292 monotherapy in patients with advanced solid tumors resulted in dose dependent peripheral Treg depletion [71]. A clinical trial of RG6292 in combination with atezolizumab is underway.

### Stability of melanoma cell-surface PD-L1 as rate-limiting to therapeutic resistance development

Various therapeutic strategies have been investigated to overcome MAPK inhibitor resistance. These include combining a type II RAF inhibitor with an allosteric MEK inhibitor and triplet combination of BRAF inhibitor plus MEK inhibitor plus anti-PD-1.

Type II RAF inhibitor plus MEK inhibitor combination prevents and overcomes acquired MEK inhibitor resistance [72]. Switching from type I to type II RAF inhibitor overcame the growth of resistant sub-lines, but only when an allosteric MEK inhibitor was present. This may be due to the superior ability of a type II RAF inhibitor plus MEK inhibitor to physically stabilize RAF/MEK and uncouple MEK/ERK. Type II RAF inhibitor plus MEK inhibitor may also have a favorable T-cell impact that supports combination with anti-PD-1/L1 therapy.

An anti-PD-1/L1 lead-in period before MAPK inhibitor combination optimizes response duration through the promotion of pro-inflammatory macrophage polarization and clonal expansion of IFN-γ high and CD8 + cytotoxic and proliferative T cells in murine models of melanoma driven by BRAF V600, NRAS, or Nf1 mutations, as well as colorectal and pancreatic carcinoma driven by KRAS G12C [73]. SequencingAnti-PD-1/L1 monotherapy before being combined with a MAPK inhibitor also reduces melanoma brain metastases and improves survival with robust T cell clonal expansion in both intracranial and extracranial metastases.

In recent years, there has been increasing rationales for immune strategies to overcome MAPK resistance. Clinical melanoma that acquires MAPK inhibitor resistance have reduced cytolytic T cells and antigen presentation [74]. Signatures of MAPK inhibitor-treated melanoma are associated with innate anti-PD-1 resistance [75]. Early on during MAPK inhibitor treatment, melanomas undergo dedifferentiation/quasi-mesenchymal transition and upregulate tumor cell surface PD-L1/L2 [76 Cancer stemness or quasi-mesenchymal phenotype is associated with abundant PD-L1, immunosuppression, metastases, and therapy resistance in general. In murine melanoma models of BRAF and NRAS mutant melanoma, high TMB increases MAPK inhibitor response durability in a CD8 + T cell dependent manner [72, 73]. Investigation of the effect of MAPK inhibitor-elicited melanoma cell surface accumulation of PD-L1/L2 on MAPK inhibitor resistance is underway. An ideal MAPK co-target would be a negative regulator of PD-L1 tumor cell surface accumulation during MAPK inhibitor therapy. Activating this negative regulator of PD-L1 should in principle suppress acquired MAPK inhibitor resistance.

### Strategies to overcome adaptive resistance to MAPK-directed therapies in melanoma

MAPK-directed therapies are often limited by adaptive resistance, frequently mediated by activation of RTK signaling and rebound of ERK activity. Combinatorial targeting of ERK signaling and SHP2, a phosphatase that mediates RAS activation downstream of multiple receptor tyrosine kinases, prevents adaptive resistance in defined subsets of ERK-dependent tumors. SHP2 inhibition overcomes feedback MAPK reactivation in BRAF V600E tumors. RAS and ERK activity are suppressed by SHP2 inhibitor in RAS G12X cells, but not in RAS G13D or RAS Q61X-expressing cells [77 SHP2 inhibition is unable to overcome MAPK rebound when RAS Q61X is expressed. RAS Q61X mutation or low p(Y542)SHP2 are negative biomarkers of response in overcoming adaptive resistance to MAPK inhibitors by co-targeting SHP2. RAS Q61X is probably the most challenging of the RAS mutants against which to direct targeted therapy and so a parallel focus on targeting effectors and adaptive response is needed.

One potential strategy to overcome adaptive drug resistance is to exploit the allosteric properties of RAF inhibitors [76]. Current RAF inhibitors preferentially bind and inhibit monomeric over dimeric RAF, meaning they selectively inhibit BRAF V600E in tumors, which can signal as a monomer, but not BRAF wild type which signals as an obligatory dimer. The inability of these RAF inhibitors to inhibit dimeric BRAF is the basis of their broad therapeutic window but is also responsible for adaptive drug resistance in which relief of negative feedback upon MAPK pathway inhibition results in rapid formation of RAF dimers. Negative allostery caused by the ‘OUT’ position of the αC-helix is the structural basis of inhibitor failure to suppress RAF dimers; vemurafenib, dabrafenib and encorafenib are all αC-OUT RAF inhibitors. RAF dimer (or ‘pan-RAF’) inhibitors suppress signaling and growth in BRAF wild-type cells but have a low therapeutic index. A third group of RAF inhibitors are selective for dimeric BRAF V600E; these include the FDA-approved multi-kinase inhibitor regorafenib, as well as LHX254 and belvarafenib (GDC-5773) that are in clinical development. These RAF inhibitors have higher inhibitor stability in the dimer specificity pocket due to stabilization of the αC helix upon RAF dimerization.

The triple combination of a RAF dimer-selective, a RAF monomer-selective, and a MEK inhibitor (regorafenib plus dabrafenib plus trametinib) was highly effective and well tolerated in multiple BRAF V600E tumor models in vivo. This triple combination was also less toxic than dimer RAF inhibitor plus MEK inhibitor. In a patient with stage IV BRAF V600E colorectal cancer that progressed on standard therapies, off-label use of this triple combination was well tolerated and effective, achieving tumor control for almost 8 months.

Moving forward in melanoma, adding a feedback inhibitor, such as SHP2, may improve outcomes in BRAF V600E-mutant patients, as may the combination of a RAF dimer inhibitor plus MEK inhibitor in RAS Q61X-mutant melanoma. The addition of SHP2 in BRAF wild-type/NRAS wild-type should also be investigated. Optimal sequencing of MAPK-targeted therapy with immunotherapy is also needed.

### Tumor metabolism in an age of cancer immunology: deconvoluting diverse metabolic programs

A founding observation in cancer metabolism was that tumors consume glucose to produce lactate in the presence of oxygen, a process known as Warburg metabolism. Tumor-infiltrating immune cells Teffs and inflammatory myeloid cells all use glycolysis; however, it is unclear which cell subsets uptake more glucose in the TME.

Glucose uptake was measured among specific cell subsets in the TME using ^[18F]^fluorodeoxyglucose (FDG)-PET imaging [77]. The most capacity for intra-tumoral glucose uptake was observed in myeloid cells, with equivalent levels of glucose uptake in tumor-infiltrating T cells and cancer cells across a range of cancer models. High glucose uptake was seen in CD11b + cells, including in B- and T cell-deficient models. This indicates that high glucose uptake in myeloid cells is independent of adaptive immunity. In addition, these data show that glucose is available in the TME and preferentially partitions into infiltrating immune cells versus cancer cells.

In contrast, cancer cells, demonstrated the highest glutamine uptake. This nutrient partitioning was cell-intrinsically programmed through mTORC1 signaling and glucose and glutamine-related gene expression. Glucose-related gene sets are enriched in myeloid cells, while fatty acid and amino acid-related pathways are enriched in cancer cells. Inhibiting glutamine uptake enhanced glucose uptake across tumor resident cell types, demonstrating that glutamine metabolism suppresses glucose uptake without glucose being limiting in the TME. Thus, cell-intrinsic processes drive the preferential immune and cancer cell uptake of glucose and glutamine, respectively.

In cancer, glucose uptake and the production of lactate increased even in an aerobic state and the presence of functioning mitochondria. This metabolic change provides substrates required for cancer cell proliferation and division, which is involved in tumor growth, metastatic progression, and long-term survival. Despite extensive research on cancer metabolism with promising results generated in the last decades, questions are still arising. Increased understanding of cancer metabolism suggests that patient FDG-PET scans need re-interpretation and new 18F-nutrient tracers could be implemented for early detection/therapeutic response. Intrinsic cell-programmed nutrient partitioning is an alternative model to nutrient competition in tumors, where diverse cell populations preferentially acquire distinct metabolites from a common pool of metabolites available in the TME. In this model, glycolytic tumors are immunoinhibitory because of large-scale microenvironmental changes that alter intrinsic cellular programming rather than directly due to nutrient deficiencies. Targeting of cell selective partitioning of these nutrients may lead to therapies that improve immune cell function in the TME while impairing the proliferation and metastasis of tumor cells. The metabolic switch in tumor cells and interaction with the immune system should be considered in the development of tumor-specific tracers and drugs, which should block specific pathway(s) in tumors but not normal tissues.

### Shall we continue to fight the dragon or try to kill its eggs?

Metaphorically, overt metastatic disease (nodal or distant) can be considered the ‘dragon’, whereas possible undetectable dormant disease, e.g., after surgery or after apparent complete response after therapy, represent the ‘eggs’. Treating eggs and dragons is conceptually very different. Eggs only exist as a probability after surgery and adjuvant therapy is based on perceived risk with a predefined completely hypothetical ideal duration, usually of one-year, and no way to evaluate its efficacy. Dragons are a reality that involves treating a detectable disease in adjuvant or metastatic settings, with duration of treatment defined by a direct reading of the effect.

Adjuvant trials suggest we can have an impact on the eggs, with delayed RFS in AJCC stage II-III and even monolesional stage IV patients. However, it is unknown whether eggs are destroyed or just ‘frozen’. The key question is whether it is easier to kill eggs than dragons or, in other words, is treatment more active in the adjuvant setting than in metastatic disease. Cross-over trials, such as the KEYNOTE-054 pembrolizumab trial, in which patients receive early adjuvant therapy or crossover to therapy at relapse should answer this question; however, this is not really the case since, at relapse, patients will receive the best treatment available at that time, and not necessarily the cross-over treatment.

Adjuvant ipilimumab has shown an OS benefit compared with placebo [78]. However, to date neither adjuvant PD-1 inhibition nor targeted therapy have shown a significant OS benefit, [79]. One explanation maybe that adjuvant therapy only increases RFS in less aggressive disease, which contributes little and later to survival. Thus, does adjuvant treatment perhaps only freeze the eggs of ‘good dragons’, which might respond to later treatment anyway, while unable to freeze the eggs of ‘bad dragons’?

Increased RFS and DMFS with adjuvant therapy is relevant even if OS is unchanged, since avoidance of relapse is very important for patient wellbeing, with relapse having a worse impact than adverse events on patients’ quality-of-life. In addition, disease evolution is unpredictable and so adjuvant therapy is an advisable strategy, even in the absence of evidence that the most aggressive eggs are the ones sensitive to adjuvant treatment and that such an evolution will be delayed or avoided. Finally, adjuvant therapy increased the likelihood that the patient will have better status if and when new metastatic treatments become available, a reasonable expectation in the context of dramatic progress in melanoma.

Whether treatment can be more active in the adjuvant setting than in metastatic disease may differ for targeted therapy and checkpoint inhibition. With BRAF/MEK inhibition, it may be easier to kill eggs than dragons, given that genomic resistance and cell plasticity leading to progressive cell reprogramming may have developed before later intervention at time of distant metastases. In contrast, response to anti-PD-1 therapy depends on the presence or absence of a good microenvironment and favorable cofactors which are less likely to change; thus, PD-1 immunosensitive eggs will probably give birth to PD-1 immunosensitive dragons. BRAF/MEK inhibition and PD-1 blockade may have a similar effect in the adjuvant but not the metastatic setting, where immunotherapy may be preferable. As such, BRAF/MEK inhibitor therapy may not be the best option as first-line in the metastatic setting but should be considered in the adjuvant setting for BRAF-mutated IIIA-B melanoma.

An important question is whether BRAF/MEK inhibition and anti-PD-1 therapy are active against the same or different eggs. In BRAF-mutant patients, the hazard ratios of adjuvant treatment with BRAF/MEK inhibition and anti-PD-1 are very similar but it is unclear whether treatments are active against different eggs with a similar outcome by chance, whether they are controlling the same eggs by the same mechanism, or whether some “good eggs” are easier to control irrespective of the treatment. It may be that the adjuvant effects limited to eggs that are easy to treat because of molecular characteristics and host response. The proportion of these responsive eggs may be the same whatever the AJCC stage, and it is only the respective proportion of bad eggs and patients free of eggs that is changing with AJCC stage. Interestingly, the same prognostic biomarkers (IFN-γ GEP) are playing in the same direction in adjuvant, metastatic and even neoadjuvant settings, for PD-1 and BRAF/MEK inhibition, suggesting that treatment efficacy may depend on disease aggressiveness and immune environment rather than on whether applied early or late (adjuvant or metastatic). Current adjuvant treatments may then not be in the situation to reduce the proportion of “aggressive dragons” (i.e., high lactate dehydrogenase, high kinetics, monoresistance) and improve OS, since they are probably unable to kill or control ‘bad eggs’.

In the metastatic setting, results are generally better in patients with low tumor load, i.e., ‘small dragons’. This is often considered an argument to treat melanoma as early as possible; however, it could also mean that low aggressiveness tumors are overrepresented in low tumor load, meaning better efficacy in low aggressiveness tumors, and not in earlier treatment.

Clearly, there are no eggs and dragons, just undetectable disease, and detectable metastatic disease, which represent two phases of the same disease, which itself may be aggressive or non-aggressive. Treating early is beneficial since it can improve RFS. However, the disease process may not be fundamentally altered, and it is likely that current adjuvant treatments do not prevent the most aggressive disease but instead only control less aggressive disease in patients who would have non-aggressive metastatic disease and who would respond to later therapy anyway. The next challenges are to find better biomarkers for detecting biological aggressiveness early on, before aggressiveness become obvious at the metastatic stage, and to develop adjuvant therapies able to overcome most of these early aggressive diseases, which may not yet be the case with PD1 and BRAF/MEK inhibition.

## What will we do in the clinic next?

### Question 1: If I choose immunotherapy for metastatic melanoma, will it be as monotherapy or combination?

In the phase III CheckMate 067 trial, median OS at a minimum follow-up of 6.5 years was 72 months with combination nivolumab plus ipilimumab, 37 months with nivolumab and 20 months with ipilimumab alone [80]. No new safety signals were observed, and no additional treatment-related deaths were reported since the 36-month follow-up. Median treatment-free interval (the time from the last dose of study drug to initiation of subsequent systemic therapy or the last known date alive) was 28 months with the combination and two months in both monotherapy arms, and 77%, 69%, and 43% of patients who were still alive were treatment-free in the three groups, respectively. Thus, combination therapy with anti-CTLA-4 and anti-PD-1 will be the preferred first choice first-line immunotherapy for most patients.

### Question 2: For a metastatic melanoma patient with a BRAF mutation, what will I choose first?

Three targeted BRAF/MEK inhibitor combinations are now available (dabrafenib plus trametinib, vemurafenib plus cobimetinib, and encorafenib plus binimetinib), and all appear fairly similar in efficacy. PD-1 blockade is also effective in patients with BRAF-mutant melanoma; 6.5-year OS rates in CheckMate 067 were 57%, 43%, and 25% in patients with BRAF-mutant tumors and 46%, 42%, and 22% in those with BRAF-wild-type tumors in the combination, nivolumab, and ipilimumab groups, respectively. There is also the potential of combining targeted therapy and immunotherapy, although results with trials of triplet regimens have not been convincing. Sequencing of treatment is also important, with nivolumab plus ipilimumab first achieving superior 2-year OS compared with initial treatment with the BRAF/MEK inhibitor regimen of dabrafenib plus trametinib (72% vs 52%; p = 0.0095) in the phase III DREAMseq trial [81]. Data from the phase II SECOMBIT trial also suggest immunotherapy with ipilimumab plus nivolumab before encorafenib plus binimetinib is associated with better outcomes than vice versa [82]. A ‘sandwich strategy consisting of encorafenib plus binimetinib for 8 weeks followed by ipilimumab plus nivolumab for 8 weeks also proved more effective than starting with targeted therapy and switching to immunotherapy at disease progression. Thus, in BRAF-mutant patients, combination immunotherapy appears to be a better first-choice option than targeted therapy, with sandwich therapy an interesting approach for symptomatic patients.

### Question 3: What will I use as adjuvant therapy for high-risk patients?

Both the KEYNOTE-054 trial of pembrolizumab versus placebo in resected stage III melanoma and the CheckMate-238 trial of nivolumab versus ipilimumab in resected stage IIIB–IV melanoma, have shown a RFS benefit of anti-PD-1 therapy [83, 84]. The COMBI-AD trial also reported a similar RFS benefit with dabrafenib plus trametinib versus placebo in patients with BRAF-mutant melanoma [79]. There is no clear first choice based on efficacy, but side effects tend to favor immunotherapy. Thus, the choice between anti-PD-1 or BRAF/MEK combination for a BRAF-mutant patient is largely a clinical decision. However, possibly the results of the DREAM-Seq trial favor adjuvant immunotherapy.

To help address these questions, mature data from SECOMBIT are required. Options for patients after failure of immunotherapy and targeted therapy need to be further investigated, and more clarity on relatlimib plus nivolumab versus combination immunotherapy as a first-line option is needed.

## Conclusion

The use of immunotherapy and targeted BRAF and MEK inhibitors therapy to treat advanced melanoma has improved clinical outcomes for many patients. Increased understanding of the TME and immune response together with increasing real-world experience with these agents is leading to greater awareness of their strengths but also existing unmet clinical needs. Primary and acquired resistance to treatment remains a major challenge. Emerging strategies are needed for these patients, including new agents, new combinations and new adjuvant or neoadjuvant approaches. There is also a critical need to develop clinically actionable biomarkers, as results from such studies may help develop a new paradigm for immune monitoring in the setting of immune checkpoint blockade with emphasis placed on assessment of an adaptive immune response in an early on treatment using liquid biopsy in addition to pretreatment markers.

## Data Availability

Not applicable.

## Abbreviations

AJCC: American Joint Committee on Cancer
APM: Antigen-processing machinery
CAF: Cancer-associated fibroblasts
CAR: Chimeric antigen receptor
cfDNA: Cell-free DNA
CLICAL: Clinical categorization algorithm
ctDNA: Circulating tumor DNA
CT: Computed tomography
CTLA-4: Cytotoxic T-lymphocyte-associated antigen
COVID-19: Coronavirus disease 2019
DC: Dendritic cell
DFS: Disease-free survival
DMFS: Distant metastases-free survival
ECT: Electrochemotherapy
ESMO: European Society for Medical Oncology
FcyR: Fcγ receptor
FDA: Food and Drug Administration
FFPE: Formalin-fixed paraffin-embedded
FMT: Fecal microbiota transplant
GEP: Gene expression profile
GITR: Glucocorticoid-induced tumour necrosis factor receptor-related protein
HLA: Human leukocyte antigen
HNSCC: Head and neck squamous cell carcinoma
IFN: Interferon
Ig: Immunoglobulin
IL: Interleukin
irAE: Immune-related adverse event
JAK: Janus kinase
LAG-3: Lymphocyte-activation gene-3
LDH: Lactate dehydrogenase
MAPK: Mitogen-activated protein kinase
MDSC: Myeloid-derived suppressor cell
MHC: Major histocompatibility complex
MPR: Major pathological response
MRI: Magnetic resonance imaging
MSS: Melanoma-specific survival
NCD: Non-communicable disease
NSCLC: Non-small-cell lung cancer
ORR: Objective response rate
OS: Overall survival
pCR: Pathological complete response
PD-1: Programmed death-1
PD-L1: Programmed death ligand-1
PET: Positron emission tomography
RECIST: Response Evaluation Criteria in Solid Tumors
RFS: Relapse-free survival
SARS‐CoV‐2: Severe acute respiratory syndrome coronavirus 2
SCC: Squamous cell carcinoma
ScFv: Single-chain fragment variable
SLNB: Sentinel lymph node biopsy
TCGA: The Cancer Genome Atlas
Teffs: T effector cells
TIM-3: T cell immunoglobulin and mucin-domain containing-3
T-Ikzf2: Terminal effector CD8 cells
TMB: Tumor mutation burden
TME: Tumor microenvironment
TNF: Tumor necrosis factor
Tregs: Regulatory T cells
VISTA: V-domain immunoglobulin suppressor of T cell activation
